# Vernier effect using in-line highly coupled multicore fibers

**DOI:** 10.1038/s41598-021-97646-0

**Published:** 2021-09-15

**Authors:** Natanael Cuando-Espitia, Miguel A. Fuentes-Fuentes, Amado Velázquez-Benítez, Rodrigo Amezcua, Juan Hernández-Cordero, Daniel A. May-Arrioja

**Affiliations:** 1 CONACyT, Applied Physics Group, DICIS, University of Guanajuato, 368850 Salamanca, Guanajuato Mexico; 2 Universidad Tecnológica de Aguascalientes, Blvd. Juan Pablo II 1302 Exhacienda la Cantera, 20200 Aguascalientes, Mexico; 3 Instituto de Ciencias Aplicadas y Tecnología, Universidad Nacional Autónoma de México, Circuito Exterior S/N, Ciudad Universitaria, 04510 Mexico City, Mexico; 4 CREOL, The College of Optics & Photonics, The University of Central Florida, Orlando Florida, 32816-2700 USA; 5 Instituto de Investigaciones en Materiales, UNAM, AP 70-360, Cd. Universitaria, 04510 Mexico City, Mexico; 6 Centro de Investigaciones en Óptica A.C., Prol. Constitución 607, Fracc. Reserva Loma Bonita, 20200 Aguascalientes, Mexico

**Keywords:** Optical sensors, Optics and photonics, Imaging and sensing

## Abstract

We demonstrate optical fiber sensors based on highly coupled multicore fibers operating with the optical Vernier effect. The sensors are constructed using a simple device incorporating single-mode fibers (SMFs) and a segment of a multicore fiber. In particular, we evaluated the performance of a sensor based on a seven-core fiber (SCF) spliced at both ends to conventional SMFs, yielding a versatile arrangement for realizing Vernier-based fiber sensors. The SMF–SCF–SMF device can be fabricated using standard splicing procedures and serve as a “building block” for both, reflection and transmission sensing configurations. As demonstrated with our experimental results, the Vernier arrangements can yield a ten-fold increase in sensitivity for temperature measurements compared to a conventional single SMF–SCF–SMF device, thereby confirming the enhanced sensitivity that can be attained with this optical effect. Furthermore, through theoretical analysis, we obtain the relevant parameters that must be optimized in order to achieve an optimal sensitivity for a specific application. Our findings thus provide the necessary guidelines for constructing Vernier-based sensors with all-fiber devices based on highly coupled multicore optical fibers, which constitutes an ideal framework to develop highly sensitive fiber sensors for different applications.

## Introduction

The optical Vernier effect is an efficient yet simple method widely used for enhancing the sensitivity of fiber optic sensors (FOS)^1,2^. Essentially, the effect is produced upon combining two interferometric or resonant structures designed with similar free spectral ranges (FSRs)^3^. When the spectral responses of both structures are superimposed, a low spatial frequency envelope is generated, and any spectral shift in one of the optical structures results in an enhanced shift of the envelope’s frequency. In practical applications, one of the structures is used as a reference while the other is allowed to be affected by an external disturbance^4^. In principle, the Vernier effect can increase the sensitivity of FOS considerably, and devices based on this effect are typically engineered by optimizing the available linewidth for detection^2^. For example, a Vernier effect-based fiber refractometer has been recently reported with a sensitivity of 500 µm/RIU, a record value for this kind of fiber sensor^5^. Thanks to the inherent advantages of fiber optics, the Vernier effect has been successfully used in temperature^6–29^, pressure^30–35^, refractive index^36–44^, strain^45–52^, curvature^53,54^, displacement^55^, and humidity^56^ FOS. Moreover, mature technologies such as focused ion bean and femtosecond laser micromachining^35,48^, photonic crystal fibers^7,36^, and fiber tapering^4,43,46^ have been effectively used in the construction of highly-sensitive Vernier devices, thus demonstrating the versatility of Vernier-based sensor schemes.

In terms of interferometric structures, Vernier-based FOS have typically utilized Fabry–Perot interferometers^1,3,6–20,34–38,45–48,55,56^, Mach–Zehnder interferometers^29–32,42,51,53^, Sagnac interferometers^21–25,39,50^ and fiber-optic ring resonators^28,40,41,52^, either connected in parallel or in tandem configurations, depending on the application. Although interferometric arrangements have been the preferred choice for generating the optical Vernier effect, a similar spectral response can be obtained from coupled optical structures. Few reports have been published on exploring highly-coupled optical devices in Vernier-based FOS^4,43^, and the advent of novel optical fibers may offer new possibilities for developing innovative sensing configurations. For example, multicore fibers (MCFs) have emerged as a promissory solution for increasing the amount of information transmitted in a single fiber; they have been studied extensively for many years and have become a mature technology with multiple core counts in a single fiber^57,58^. These specialty fibers have also been used in FOS due to their ability to transmit multiple signals, an advantageous feature for applications such as tridimensional deformation detection^59,60^. Despite their attractive features for sensing applications, only one work has explored the use of MCFs for implementing the Vernier effect^54^, albeit using an MCF with negligible coupling effects due to a large separation among the cores (24 μm). While uncoupled cores allow for increasing the number of individual channels within a fiber, coupled MCFs generate multiple spatial light distributions, modes or super-modes^61,62^. Highly-coupled cores also provide multimode interference effects and energy transfer within cores, thus yielding a wavelength modulated transmission spectrum whose features depend on the physical parameters of the MCF^58^. For example, highly-coupled seven core fibers (SCFs)^63,64^ allocate the cores symmetrically within the structure yielding a sinusoidal spectral response, very similar to that obtained from interferometric arrangements^58,65^. These spectral features of SCFs have also been demonstrated to be remarkably sensitive for sensing applications^63,66^.

In this work, we demonstrate the implementation of the optical Vernier effect using solely highly-coupled SCFs as an alternative to conventional interferometric arrangements. The fundamental difference for generating the spectra arising from the Vernier effect lies in the fact that a small length of a few centimeters of a SCF can effectively generate a sinusoidal spectral response useful either as a reference or a sensing signal. Among other advantages, the proposed approach requires an exceptionally undemanding fabrication process, provides excellent versatility for transmission and reflection sensing schemes, is fully compatible with conventional fiber technologies, and shows excellent potential for being incorporated in different processes for further sensitivity enhancement and straightforward analysis. Thus, as shown in the following sections, our proposal results in a simple method to fabricate highly sensitive fiber sensors without the need for sophisticated and/or expensive fabrication approaches.

### Principle of operation

The building block for a sensor based on an SCF comprises two conventional single-mode fibers (SMFs) spliced at both ends of an SCF section. A highly-coupled MCF such as the SCF used in this work can be modeled using coupled-mode theory^61,62,67,68^, and for an SMF–SCF–SMF arrangement, the normalized intensity can be shown to be (see Methods for details):

1 $$I=\frac{1}{7}\left\{3\mathrm{cos}\left(2\sqrt{7}\kappa L\right)+4\right\}.$$

**Figure 1:**
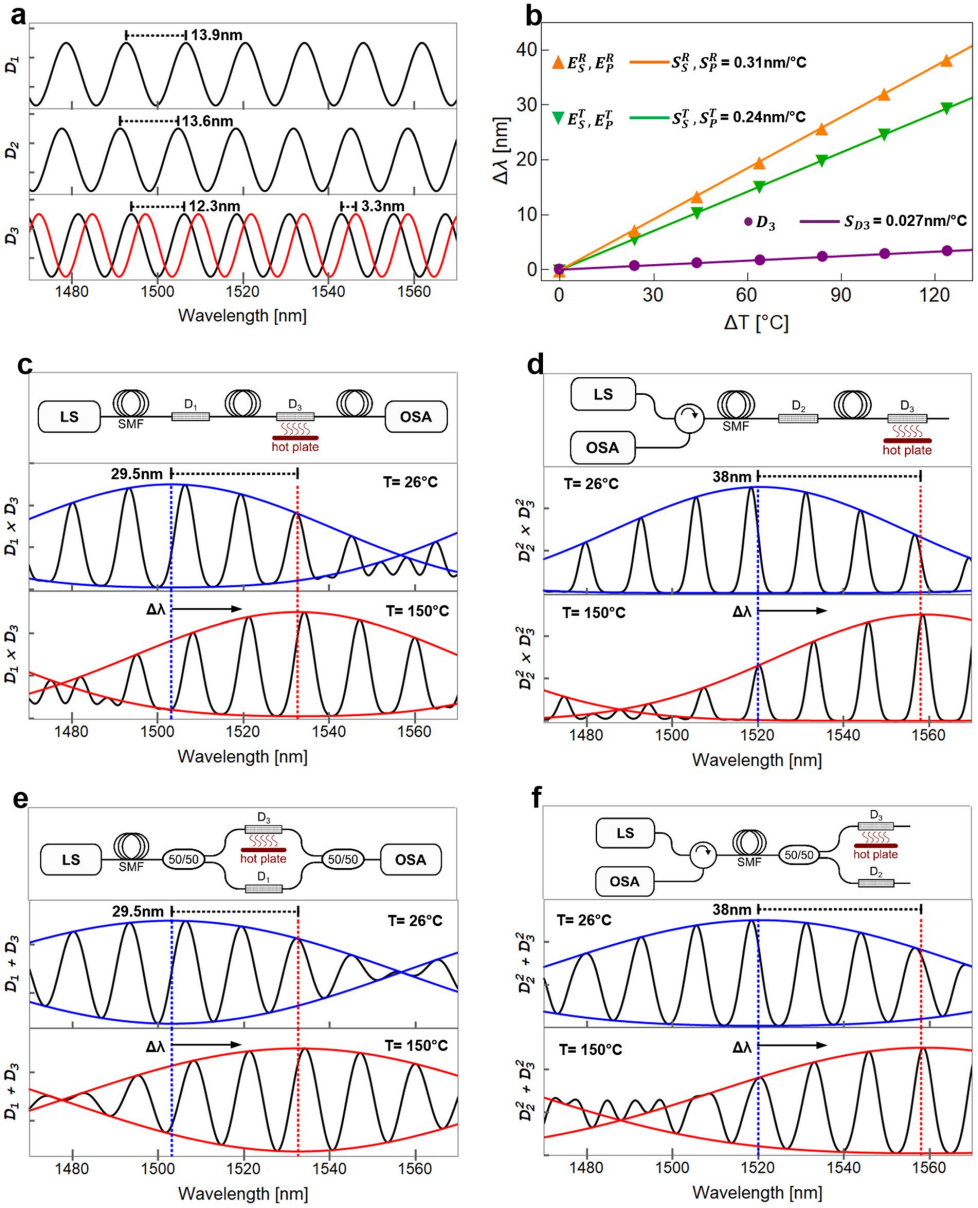
(**a**) Simulated spectral response at room temperature (26 °C) of SCFs with different lengths (solid black lines) and spectral response of device D_3_ at 150 °C (solid red line), (**b**) Simulated spectral shift as a function of the applied temperature for device D_3_ and also for the different Vernier configurations, (**c**)–(**f**) Vernier configurations and their simulated spectral responses at room temperature (26 °C) and heated at 150 °C.

In this expression, *I* is the normalized intensity, *L* is the length of the SCF, and *κ* represents the coupling coefficient, which is, in general, a function of the optical and geometrical features of the SCF. Fig. 1a shows in solid black curves the spectral features of the normalized intensity calculated by means of Eq. (1) for three SMF–SCF–SMF devices (*D*_*1*_, *D*_*2,*_ and *D*_*3*_) with a fixed coupling coefficient (*κ*) and different SCF lengths (*L*_*1*_ = 9.30, *L*_*2*_ = 9.57 and *L*_*3*_ = 10.5 cm, respectively). The coupling coefficient *κ* was evaluated using core and cladding refractive index of *n*_*co*_ = 1.45 and *n*_*cd*_ = 1.444. Core diameters of 2*ρ* = 9 μm and a center-to-center core separation of *d* = 11.86 μm were obtained using image processing; these dimensions are within the typical values reported for this type of highly-coupled MCFs^59,60,63–66^. Briefly, we high-contrasted the original image of the SCF, applied edge detection and binarization to find the area of each core. The mass centroid of these areas were set as the cores centers and the resulting areas were used to find an equivalent disk with the same area. The mean value of the seven equivalent disks was 9.0 μm. Similarly, the mean value between mass centroids was 11.86 μm. As seen in Fig. 1a, the transmission spectra of the three devices show similar FSRs (13.9, 13.6, and 12.3 nm, respectively), a key requirement for implementing the Vernier effect. To illustrate the impact of an external disturbance on the spectral response of an SMF-SCF-SMF device, we have simulated a temperature increase in device *D*_*3,*_ and the result is shown in the bottom plot of Fig. 1a as a solid red curve. The simulated temperature increment was from 26 to 150 °C, accounting for changes in the refractive indices of the SCF according to the thermo-optic coefficients of the core (9.75 × 10^−6^ °C^−1^) and cladding (9.5 × 10^−6^ °C^−1^) materials, as previously reported^69,70^. As depicted in Fig.1a, the increase in temperature shifts the spectrum by ~ 3 nm, which agrees well with the reported sensitivity of 28.7 pm°C^−1^ using similar SCF structures^65^.

Let us now consider the possible configurations of SMF–SCF–SMF devices that may serve for implementing the Vernier effect. We have recently shown that the spectral response of such devices connected in series and parallel can be effectively modeled by the product and addition, respectively, of their individual responses^66^. Furthermore, and in contrast with most interferometric fiber structures, SCF-based fiber devices can be readily implemented in both transmission and reflection configurations. As a result of the versatility of the SMF–SCF–SMF building blocks, four basic arrangements for implementing the Vernier effect can be derived; namely: readout from the transmission spectrum of two devices connected in series or parallel (i.e., transmission-series and transmission-parallel) and readout from the back-reflected spectrum from the devices again connected in either way (i.e., reflection-series and reflection-parallel). As shown schematically in Fig. 1c–f, these configurations can be realized with standard fiber optic devices such as 50/50 couplers and optical circulators, and their spectral features may be obtained upon launching a broad-band light source (LS) and registering either the reflected or the transmitted signals using an optical spectrum analyzer (OSA). While the spectral envelope of the concatenated SMF–SCF–SMF devices can be obtained as the product of their individual spectra, the combined signal from the parallel configuration can be calculated as the addition of each spectrum (see details in the Methods section). The analytical expressions of the spectral envelopes are thus given by:

2 $${E}_{ser}^{Tx}=\frac{1}{98}\left\{41\pm 48\mathrm{cos}\alpha +9\mathrm{cos}2\alpha \right\},$$

3 $${E}_{par}^{Tx}=\frac{1}{14}\left\{8\pm 6\mathrm{cos}\alpha \right\},$$

4 $${E}_{ser}^{Rx}=\frac{1}{{98}^{2}}{\left(41\pm 48\mathrm{cos}\alpha +9\mathrm{cos}2\alpha \right)}^{2},$$

5 $${E}_{par}^{Rx}=\frac{1}{98}\left\{41\pm 48\mathrm{cos}\alpha +9\mathrm{cos}2\alpha \right\}.$$

In Eqs. (2)–(5), we have denoted the envelope of the transmission and reflection configurations as ${E}^{Tx,Rx}$ and the envelopes for the series and parallel configurations are denoted as ${E}_{ser,par}$, respectively. In addition, we used $\alpha =\sqrt{7}({{\kappa }_{s}L}_{s}-{\kappa }_{r}{L}_{r})$ where the subscripts *s* and *r* refer to sensing and reference devices, respectively. The subscript in the coupling coefficient *κ* implies that this factor may vary independently for each fiber device, for instance, due to external disturbances such as temperature. Notice that Eqs. (2)–(5) contain a term with a (±) sign that corresponds to the upper and lower envelope for each case. Also, note that the expressions show that the coefficient for the fundamental component (cos *α*) is larger than that of its harmonic counterpart (cos 2*α*). Hence, the former dominates in the spectral envelopes obtained with the configurations studied here. This last condition implies that for the same value of *α,* the maximum of the envelope occurs at the same wavelength regardless of the configuration. Moreover, the maximum of the envelope may change with variations in one of the coupling coefficients of the SCFs, or by implementing the Vernier configuration with different lengths of SCF in one of the fiber devices.

We used Eqs. (2)–(5) to simulate the spectral response for each configuration, namely, two transmission arrangements using devices *D*_*1*_ and *D*_*3*_ (Fig. 1c,e), and two reflection schemes using devices *D*_*2*_ and *D*_*3*_ (Fig. 1d,f). The resulting spectra are shown as solid black curves in the corresponding figure; these were obtained using the same parameters (i.e., refractive indices, core sizes, and core separations) considered for obtaining the spectral response of a single SMF–SCF–SMF device (Fig. 1a). It is evident from the figures that the resulting spectra display a sinusoidal-like behavior with a very similar periodicity to that of the spectrum of the single device. Notice, however, that in contrast to the latter spectra, the arrangements connected in series and parallel, either for the transmission or the reflection configurations, exhibit a low-frequency modulation envelope characteristic of the Vernier effect. For clarity, the upper and lower envelopes of the modulated signals at a reference and sensing temperature of 26 °C are calculated using Eqs. (2)–(5), and are also included in the figures represented as solid blue curves.

The temperature sensing performance of the SMF–SCF–SMF operating on the Vernier arrangements was also explored using Eqs. (2)–(5). We chose *D*_*3*_ as the sensing element for these calculations while the others remained unperturbed, serving as reference elements. A temperature increment from ~ 26 °C (room temperature) to 150 °C was used for the calculations. The changes in the refractive indices of the core and cladding materials of the SCF used in *D*_*3*_ were obtained considering their corresponding thermo-optic coefficients. The normalized spectra (solid black curves) for the maximum temperature (i.e., T = 150 °C) along with their corresponding envelopes (solid red curves) are shown in the bottom plots of Fig. 1c–f. As seen in the figures, the increase in temperature produces a spectral shift towards longer wavelengths. While the periodic-like behavior of the modulated spectra is preserved, the maxima of the envelopes are clearly red-shifted by Δλ, as indicated in the figure. For both transmission configurations (Fig. 1c,e), the wavelength shift is the same (Δλ = 29.5 nm); similarly, both reflection configurations (Fig. 1d,f) yield the same wavelength shift (Δλ = 38 nm). The spectral positions of the envelopes’ maxima were obtained for different temperatures yielding the plot in Fig. 1b, showing that the spectral shift (Δλ) increases linearly with temperature for all the configurations. Notice that the linear fittings for the two reflection configurations yield the same slope ($${S}_{ser}^{Rx}={S}_{par}^{Rx} $$**=** 0.31 nm°C^−1^), as is also the case for the transmission arrangements ($${S}_{ser}^{Tx}={S}_{par}^{Tx}=$$ 0.24 nm°C^−1^). Interestingly, despite using the same device for sensing (*D*_*3*_), the reflection configuration provides an improved sensitivity compared to that of the transmission arrangement. This improvement is because the SCF lengths considered for *D*_*2*_ and *D*_*3*_ were more akin than those of *D*_*1*_ and *D*_*3*_ (recall that *L*_*1*_ = 9.30, *L*_*2*_ = 9.57, and *L*_*3*_ = 10.5 cm). For comparative purposes, we have included in Fig. 1b the calculated wavelength shift over the same temperature range for the individual sensing device *D*_*3*_, yielding a sensitivity of $${S}_{D3}=$$ 0.027 nm°C^−1^. The Vernier configurations therefore provide enhancement factors close to 9 and 11 times in sensitivity compared to the single sensing device.

## Experimental results

**Figure 2:**
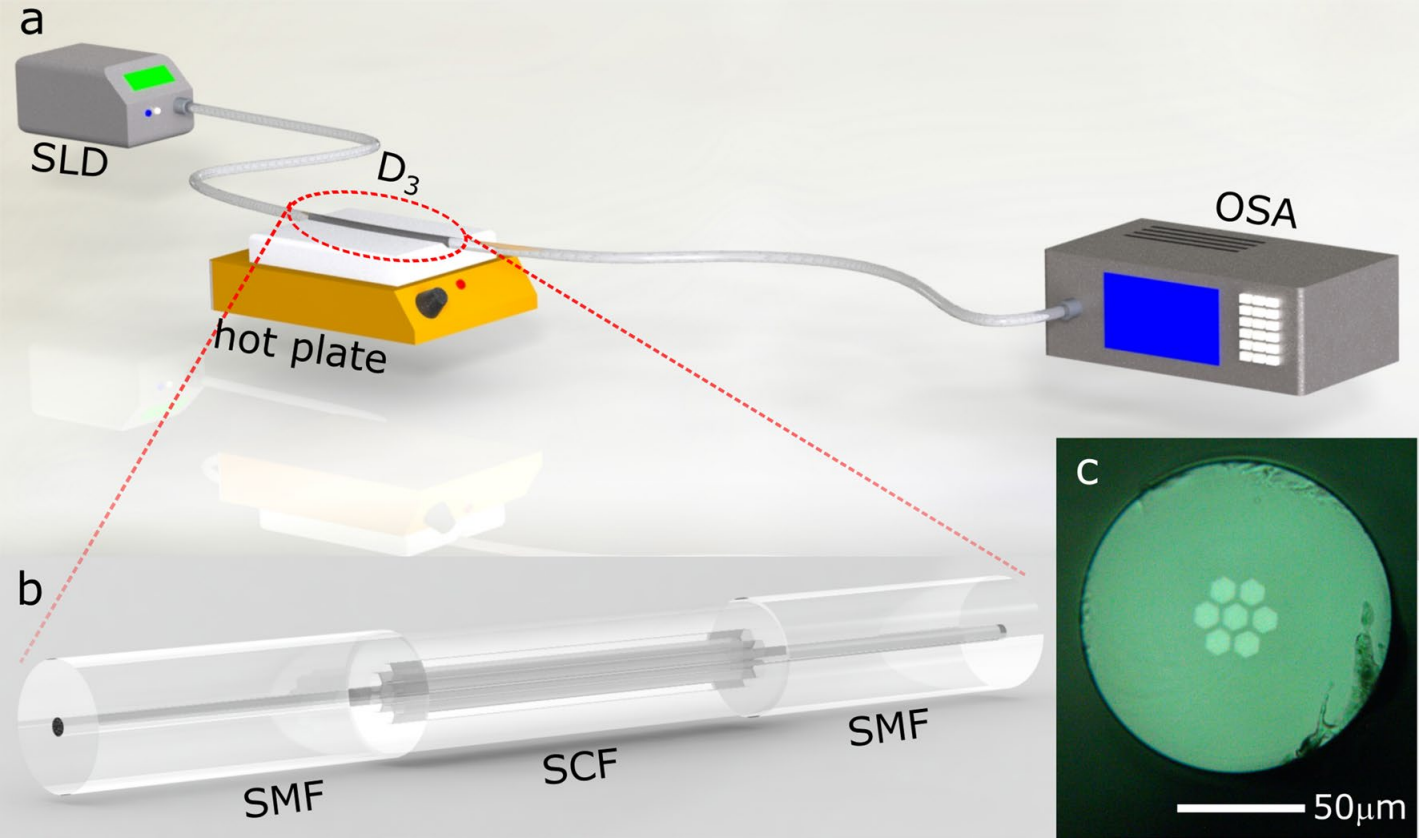
Schematic representation of the basic structure of an SMF–SCF–SMF device. The figure also shows the experimental setup used to evaluate the temperature sensing performance of the device, as well as a photograph of the cross-section of the SCFs used to fabricate the devices.

For experimental validation, we first fabricated three individual SMF–SCF–SMF devices by splicing commercial SMF (Corning SMF-28e) on both ends of a highly-coupled hexagonal-core SCF as depicted in Fig. 2b. The SCF used in our experiments was manufactured by the Microstructured Fibers and Devices group at CREOL-UCF^63^. This fiber has a symmetric core distribution in a hexagonal array, and the cross-sections of the cores are also hexagonal (see Fig. 2c). Other relevant features of the SCF include: NA = 0.1317, 120 μm cladding diameter, equivalent core diameter 2*ρ* = 9 μm and center-to-center core separation of *d* = 11.86 μm (see principle of operation section). The difference among the three fabricated devices is the SCF length, selected as *L*_*1*_ = 9.30 cm, *L*_*2*_ = 9.57 cm, and *L*_*3*_ = 10.50 cm, for devices *D*_*1*_, *D*_*2,*_ and *D*_*3*_, respectively. It is important to note that the SCFs are known to be bend-sensitive^71^; thus, care was taken to keep the devices straight in order to avoid any disturbances due to bending.

**Figure 3:**
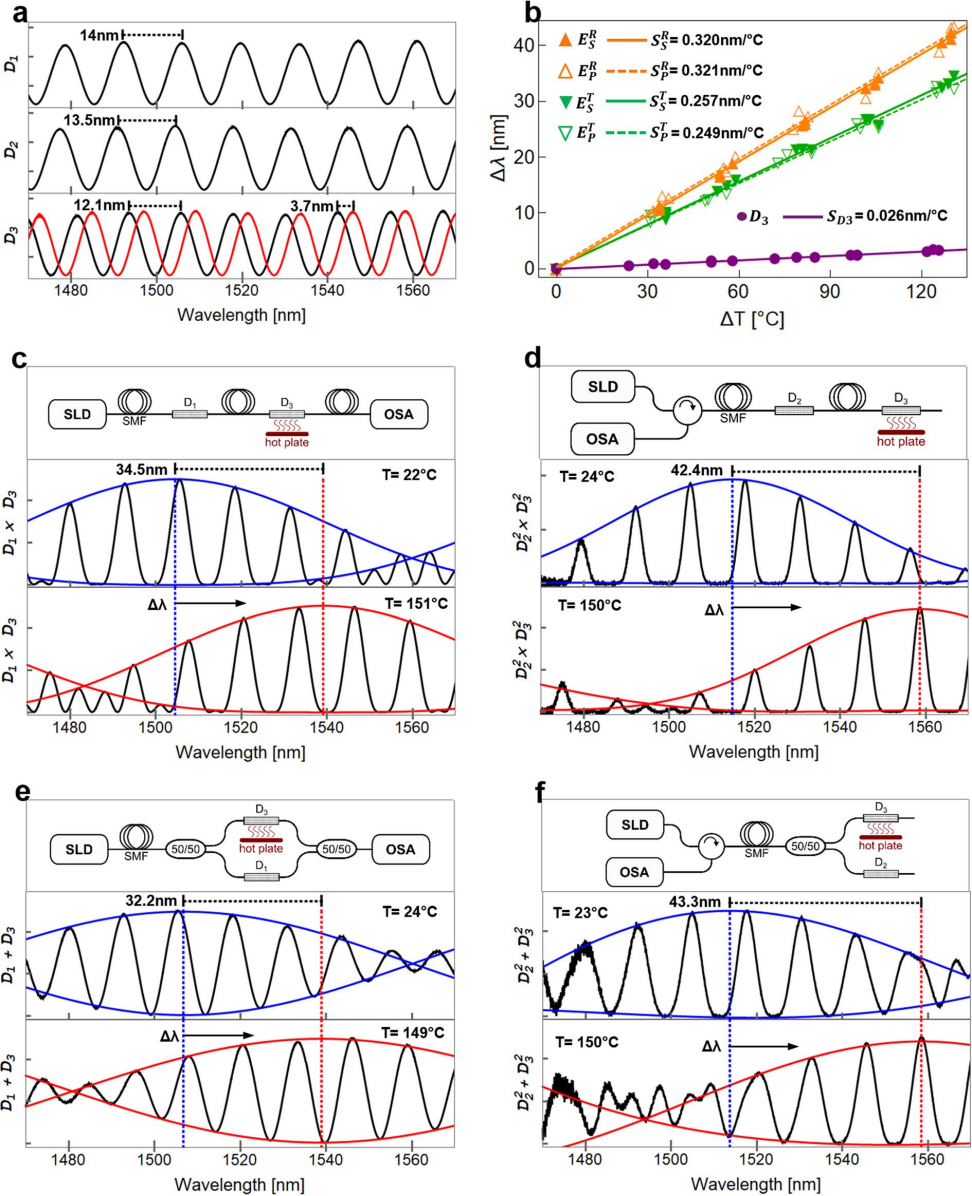
(**a**) Experimental spectral response at room temperature (26 °C) of SCFs with different lengths (solid black lines) and spectral response of device D_3_ at 150 °C (solid red line), (**b**) Experimental spectral shift as a function of the applied temperature for device D_3_ and also for the different Vernier configurations, (**c**)–(**f**) Vernier configurations and their experimental spectral responses at room temperature (26 °C) and heated at 150 °C.

The individual spectral features of the devices were obtained using a superluminescent diode (SLD) as the light source (150 nm bandwidth) and an optical spectrum analyzer (OSA) as depicted schematically in Fig 2a. The normalized spectra of the three devices are shown in Fig 3a as solid black lines, showing FSRs of 14, 13.5, and 12.1 nm, as measured with the OSA, for devices *D*_*1*_, *D*_*2,*_ and *D*_*3*_, respectively. Notice that the spectra and FSRs agree well with those obtained for the simulated devices calculated using Eqs. (2)–(5) and shown in Fig. 1a. Next, we evaluated the response to temperature changes for the individual device *D*_*3*_ upon increasing the temperature from 26 to ~ 150 °C using a ceramic hotplate. For these experiments, the polymer coating was removed from the full SCF length to avoid any contributions due to thermal expansion/elongation from the polymer, and care was also taken to maintain the fiber in contact with the surface of the hotplate at all times. The temperature was simultaneously monitored with a thermocouple placed at the center of the hotplate, and the corresponding spectra were recorded once thermal stability was achieved. The spectrum obtained for the maximum temperature (150 °C) is included at the bottom plot of Fig 3a (solid red curve). As indicated in the figure, the measured wavelength shift was 3.7 nm, which agrees with the calculated spectral shift depicted in Fig. 1a. A plot of the wavelength shift (Δλ) as a function of the temperature increase (Δ*T* = *T*_measured_ − *T*_amb_) is also included in Fig. 3b, showing a linear relation between both parameters. Notice also that the linear fit of the experimental points yields a slope that is very close to that obtained from the calculations (see Fig. 1b).

All four configurations required to obtain the Vernier effect (i.e., transmission-series, reflection-series, transmission-parallel, and reflection-parallel) were assembled using the devices *D*_*1*_, *D*_*2,*_ and *D*_*3*_. As done for the calculations shown in the previous section, we used *D*_*3*_ as the sensing element while *D*_*1*_ and *D*_*2*_ were used as reference devices. The corresponding setups are schematically depicted in the top panels of Fig. 3c–f, showing the layout of the different arrangements as well as the 50/50 couplers and the optical circulator required for their assembly. We first registered the spectra for room temperature conditions (i.e., *T* = *T*_*amb*_) as seen in the plots in the figures' middle panels. As expected, the spectra showed the low-frequency modulation envelope characteristic of the Vernier effect. For clarity, we have included in the plots the upper and lower envelopes of the modulated signals (solid blue curves) calculated using Eqs. (2)–(5). Despite the noise-like features in the reflection configuration spectra, we have an excellent fitting of the calculated envelopes with the experimental results. We then registered the spectral changes due to temperature upon heating *D*_*3*_ from 26 to 150 °C in each configuration; the spectrum obtained for the maximum temperature for each case is included in the bottom panels of the figures along with their corresponding envelopes (solid red curves) calculated by means of Eqs. (2)–(5) considering the thermo-optic coefficients of the SCF. The largest envelope spectral shift is indicated in the figures and also included in Table 1, which contains the results obtained from the calculations from the previous section. It is evident from Table 1 that the experimental sensitivities are in good agreement with the theoretical predictions. The resulting wavelength shift of the envelopes’ peaks as a function of temperature are included in Fig. 3b, showing results for three measurements for each of the tested temperatures. A fit for these points yields a linear relationship between the wavelength shift (Δλ) and the temperature increment (ΔT) for the four arrangements. Notice that as calculated from the theoretical analysis, the slope (sensitivity) obtained for the reflection configurations is larger than that of the transmission arrangements (0.32 vs. 0.25 nm°C^−1^, respectively). These features coincide again with the theoretical analysis, and the sensitivities are also in excellent agreement with the predicted values obtained from Eqs. (2)–(5). For comparison, a plot of the spectral shift as a function of temperature obtained using only *D*_*3*_ is also included in Fig. 3b, yielding a sensitivity of 0.026 nm°C^−1^. Compared to this value, the Vernier arrangements provide an enhancement in sensitivity of 12.1 and 9.6 times for the reflection and transmission configuration, respectively.

**Table 1:** Summary of studied configurations and experimental results.

Configuration		Device		$\left|\Delta L\right|$[mm]	$\left|\Delta FSR\right|$[nm]	$S$[nm °C^−1^]	
Readout	Series/parallel	Sensing	Reference			Theory	Experiment
Transmission	Series	*D*_*3*_	*D*_*1*_	12	1.9	0.24	0.257
Transmission	Parallel	*D*_*3*_	*D*_*1*_	12	1.9	0.24	0.249
Reflection	Series	*D*_*3*_	*D*_*2*_	9.3	1.4	0.31	0.320
Reflection	Parallel	*D*_*3*_	*D*_*2*_	9.3	1.4	0.31	0.321
–	–	*D*_*3*_	–	–	–	0.027	0.026

The table includes the difference in length of the SCF used for the sensing and reference devices ($\left|\Delta L\right|={L}_{S}-{L}_{R}$), as well as their differences in Free Spectral Range (i.e., $\left|\Delta FSR\right|={FSR}_{S}-{FSR}_{R}$). The sensitivities for each case are calculated as $S=\Delta \lambda /\Delta T$.

The enhancement in sensitivity obtained with the Vernier configurations is related to the so-called *M-*factor^72^, which is inversely proportional to the difference in FSRs of the devices used for the setup. In our case, the reflection configurations were constructed using *D*_*2*_ and *D*_*3*_, which have a smaller difference in FSR than devices *D*_*1*_ and *D*_*3*_, used for the transmission arrangements (see Table 1). Thus, in theory, the sensitivity of the Vernier setups may increase considerably as the FSRs (i.e., the SCF lengths) of the devices are closely matched^2^. However, increased sensitivities also require more complex detection instruments, and this must be considered for practical realizations of Vernier-based FOS. Some of these ideas will be discussed in the following sections.

## Discussion

For a given SCF, the coupling coefficient is generally a function of the wavelength and the temperature. Explicitly, it can be expressed as^67,68^

6 $$\kappa \left(\lambda ,T\right)=\frac{\sqrt{\delta }}{\rho }\frac{{U}^{2}}{{V}^{3}}\frac{{K}_{0}\left[\frac{Wd}{\rho }\right]}{{K}_{1}^{2}\left[W\right]}.$$

**Figure 4:**
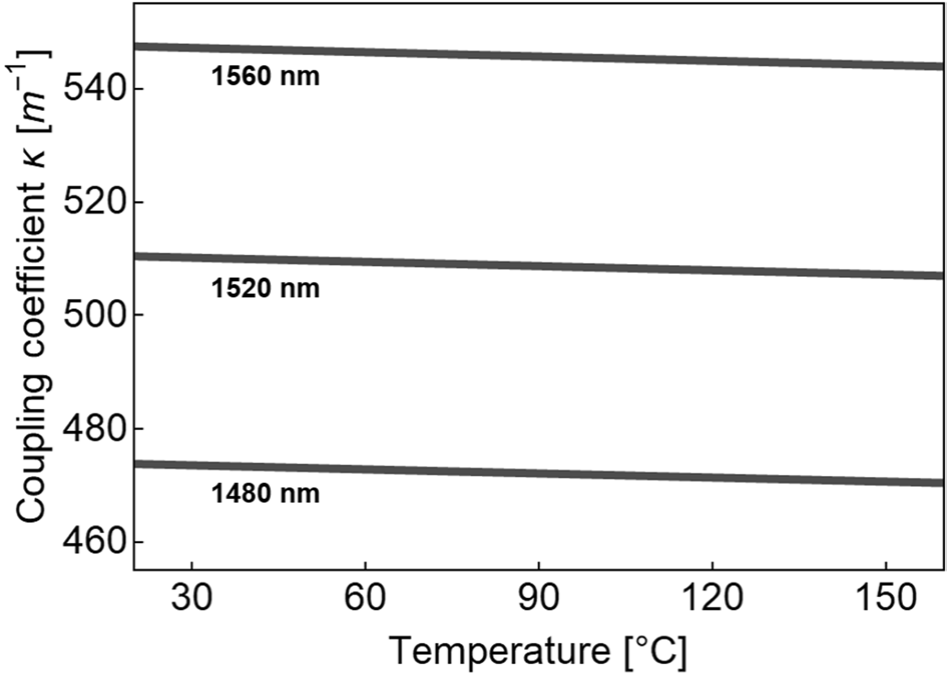
The coupling coefficient of the SCF as a function of temperature for three different wavelengths.

In this expression, the temperature-dependent refractive indices of the cladding (${n}_{cd}\left(T\right)$) and fiber core (${n}_{co}\left(T\right)$) are contained in the factor $\delta =1-{\left\{\frac{{n}_{cd}\left(T\right)}{{n}_{co}\left(T\right)}\right\}}^{2}.$ The distance between adjacent cores is represented as *d,* and $\rho$ is the core radius. ${K}_{0}$ and ${K}_{1}$ are the modified Hankel functions of order 0 and 1, respectively, whereas $U$, $V$, and $W$ are related by ${V}^{2}={U}^{2}+{W}^{2}$. The $U$ parameter can be approximated as $U=2.405{e}^{-\left(1-\frac{\delta }{2}\right)/V}$ while the $V$ parameter is defined as:

7 $${V}^{2}={\left(\frac{2\pi \rho {n}_{co}}{\lambda }\right)}^{2}\delta ,$$

where $\lambda$ is the wavelength of light in vacuum and the temperature dependence of ${n}_{co}$ has been obviated. In a previous report^66^, we have shown that for constant ambient temperature, the coupling coefficient of an SCF exhibits a linear tendency around NIR wavelengths. Similarly, using Eqs. (6) and (7) it can also be shown that for a constant wavelength, the coupling coefficient follows a linear trend in the temperature range from 20 to 160 °C, as depicted in Fig. 4.

It follows then that the intricate functional form of $\kappa (\lambda ,T)$ (Eq. (6)) can be simplified substantially by means of first-order approximations. For example, in terms of a first-order Taylor series expansion around $({\lambda }_{0},{T}_{0})$, the coupling coefficient can be expressed as:

8 $$\kappa \left(\lambda ,T\right)=\kappa \left({\lambda }_{0},{T}_{0}\right)+{\left(\frac{\partial \kappa }{\partial T}\right)}_{\left({\lambda }_{0},{ T}_{0}\right)}\left({T-T}_{0}\right)+\left[{\left(\frac{\partial \kappa }{\partial \lambda }\right)}_{\left({\lambda }_{0},{ T}_{0}\right)}+{\left(\frac{{\partial }^{2}\kappa }{\partial \lambda \partial T}\right)}_{\left({\lambda }_{0},{ T}_{0}\right)}\left({T-T}_{0}\right)\right]\left({\lambda -\lambda }_{0}\right),$$

where $\partial$ denotes the partial derivative. Assuming that the term involving the second derivative is much smaller than the first derivative term (i.e., ${\left(\frac{{\partial }^{2}\kappa }{\partial \lambda \partial T}\right)}_{\left({\lambda }_{0},{ T}_{0}\right)}\ll {\left(\frac{\partial \kappa }{\partial \lambda }\right)}_{\left({\lambda }_{0},{ T}_{0}\right)}$), the expansion now reads:

9 $$\kappa \left(\lambda ,T\right)=\kappa \left({\lambda }_{0},{T}_{0}\right)+{\left(\frac{\partial \kappa }{\partial T}\right)}_{\left({\lambda }_{0},{ T}_{0}\right)}\left({T-T}_{0}\right)+{\left(\frac{\partial \kappa }{\partial \lambda }\right)}_{\left({\lambda }_{0},{ T}_{0}\right)}\left({\lambda -\lambda }_{0}\right)=A+B\left({T-T}_{0}\right)+C\left({\lambda -\lambda }_{0}\right),$$

where: $A=\kappa ({\lambda }_{0},{T}_{0}), B={\left(\frac{\partial \kappa }{\partial T}\right)}_{\left({\lambda }_{0},{ T}_{0}\right)}, and C={\left(\frac{\partial \kappa }{\partial \lambda }\right)}_{\left({\lambda }_{0},{ T}_{0}\right)}$ are constants that can be quickly evaluated using Eq. (6). Recall from Eq. (1) that for an SMF–SCF–SMF arrangement, the argument of the sinusoidal term for the normalized intensity is $2\sqrt{7}\kappa L$; hence, using Eq. (9), the spatial frequency (in rad m^−1^) is simply $\omega =2\sqrt{7}CL$. This last equation implies that the spatial frequency depends only on the length of the SCF and does not vary with temperature, in agreement with the experimental results. Meanwhile, Eq. (9) allows for expressing the phase as $\varphi =2\sqrt{7}L\left\{A+B\left({T-T}_{0}\right)+C{\lambda }_{0}\right\}$, showing that temperature changes will only affect the phase. Upon evaluating the derivative of the phase with respect to temperature, we can obtain the sensitivity as:

$$S=\frac{\partial \varphi }{\partial T}=2\sqrt{7}BL \left[rad\cdot {^\circ C}^{-1}\right]$$

This last equation can be expressed in wavelength units using the spatial frequency, i.e.:

10 $$S=\frac{\partial \varphi }{\partial T}\cdot \frac{1}{\omega }=\frac{2\sqrt{7}BL}{2\sqrt{7}CL}=\frac{B}{C} \left[m\cdot {^\circ C}^{-1}\right]$$

To illustrate the usefulness of this approximation, let us evaluate the sensitivity for device *D*_*3*_: for an SCF length of 10.5 cm and using $({\lambda }_{0},{T}_{0})=(1520 nm,70^\circ C)$, we obtain $S=0.0269 \left[nm\cdot {^\circ C}^{-1}\right]$; notice that this is in close agreement with both, the theoretical sensitivity estimated for the device ($0.027 nm\cdot {^\circ C}^{-1}$) and the experimental result ($0.026 nm\cdot {^\circ C}^{-1}$).

The same approach can be followed to analyze the phase and frequency of the spectral envelopes obtained with the Vernier configurations. For instance, using Eq. (9), the change in phase with respect to temperature can be shown to be:

11 $${S}_{V}=\frac{\partial \varphi }{\partial T}=\frac{B}{C}\frac{{L}_{s}}{{(L}_{s}-{L}_{r})} \left[m\cdot {^\circ C}^{-1}\right]$$

where *L*_*s*_ and *L*_*r*_ are respectively the lengths of the SCFs used for the sensing and reference devices. Using the lengths for devices *D*_*1*_ and *D*_*3*_ (i.e., *L*_*1*_ = 9.30 cm and *L*_*3*_ = 10.50 cm), we get ${S}_{V}=0.236 \left[nm\cdot {^\circ C}^{-1}\right]$, in close agreement with the theoretical and experimental sensitivities included in Table 1. Similarly, using now devices *D*_*2*_ and *D*_*3*_ (*L*_*2*_ = 9.57 cm and *L*_*3*_ = 10.50 cm) the approximation yields ${S}_{V}=0.304 \left[nm\cdot {^\circ C}^{-1}\right]$, again in agreement with the values reported in Table 1 for the Vernier arrangements constructed with these devices.

Upon comparing Eqs. (10) and (11), it is clear that the sensitivity enhancement in the Vernier configurations is given by the factor:

12 $${M}_{V}=\frac{{L}_{s}}{{(L}_{s}-{L}_{r})}$$

Notice that the magnification is affected by the difference in lengths of the SCFs used for sensing and reference devices: the closest the lengths of the SCFs, the larger the enhancement in sensitivity, as observed in our experimental results. According to this expression, *M*_*V*_ could be arbitrarily large if the lengths of SCFs are matched (i.e., ${L}_{s}={L}_{r}$); however, practical considerations will limit the enhancement in sensitivity that can be achieved with the Vernier configurations. To demonstrate this, let us first evaluate in terms of the approximation the *FSR* of the Vernier arrangements, given by one-half of the period of the spectral envelope. This evaluation can be shown to be:

13 $${FSR}_{V}=\frac{\pi }{{\sqrt{7}C(L}_{s}-{L}_{r})}$$

showing that the *FSR* increases just as arbitrarily as *M*_*V*_ when the lengths of the SCFs are matched. Evidently, measuring an arbitrarily large FSR is not practical because the spectral bandwidth will be limited either by the optical spectrum analyzer or by the light source used in the experimental setup. For a given spectral bandwidth ($\Delta {\lambda }_{inst}$) and for a targeted temperature range ($\Delta {T}_{range}$), the optimum sensitivity (${S}_{opt}$) for the Vernier arrangement will be given by:

14 $${S}_{opt}=\frac{{\Delta \lambda }_{inst}}{{\Delta T}_{range}}=\frac{B}{C}\frac{{L}_{s}}{{(L}_{s}-{L}_{r})}=\frac{B}{C}{M}_{V}$$

Meanwhile, the optimum Free Spectral Range (*FSR*_*opt*_) of the spectral envelope should be fully resolved by the available spectral bandwidth, i.e.:

15 $${FSR}_{opt}={\Delta \lambda }_{inst}=\frac{\pi }{{\sqrt{7}C(L}_{s}-{L}_{r})},$$

Thus, Eqs. (14) and (15) can be used to obtain the lengths for the SCFs (*L*_*so*_ and *L*_*ro*_) required to obtain optimum performance from the Vernier configurations. From these expressions, it is straightforward to show that these lengths are given by:

16 $${L}_{so}=\frac{\pi }{\sqrt{7} B{\Delta T}_{range}} , {L}_{ro}=\frac{\pi }{\sqrt{7}}\left\{\frac{1}{B{\Delta T}_{range}}-\frac{1}{C{\Delta \lambda }_{inst}}\right\}$$

Therefore, the optimum lengths can be calculated using the constants *B* and *C* of the coupling coefficient approximation and practical considerations such as the temperature range to be measured and the spectral bandwidth available for sensor interrogation.

It is clear from Eq. (16) that the targeted temperature range will dictate the required length of the sensing device (i.e., the length for the sensing SCF). The larger the desired temperature range, the shorter the required device and vice versa. As for the length of the reference device, the bandwidth of the instruments (either the light source or the optical spectrum analyzer) for an experimental setup are typically known beforehand; thus, for a fixed value of Δλ_*inst*_, the length of the SCF can be readily calculated for the targeted temperature range. As an example, for our experiments, we have Δλ_*inst* _~ 100 nm and Δ*T*_*range*_ ~ 130 °C, yielding *L*_*so*_ = 36 cm and *L*_*ro*_ = 35cm. Evidently, these lengths are longer than those used in our devices; we should therefore expect an improved performance when increasing the lengths of the SCFs used in the Vernier configurations.

## Conclusions

Highly-coupled multicore fibers provide adequate spectral features for generating the optical Vernier effect using all-fiber configurations. We have demonstrated that optical fiber sensors based on this effect can be realized by means of a simple device based on SMFs and a segment of a multicore fiber. In particular, we evaluated the performance of a sensor based on an SCF spliced at both ends to conventional SMFs, yielding a versatile device for realizing Vernier-based fiber sensors. The SMF–SCF–SMF device was constructed following standard splicing procedures, and it was shown to serve as a “building block” for reflection and transmission sensing configurations. Hence, in contrast to other approaches using interferometric devices, our approach does not require elaborated fabrication nor high-precision adjustments on the lengths of the SCF. As demonstrated with our experimental results, the Vernier arrangements can yield a ten-fold increase in sensitivity for temperature measurements compared to a single SMF–SCF–SMF device, thereby confirming the enhanced sensitivity that can be attained with this optical effect. Through theoretical analysis of the proposed devices, we showed that the relevant parameter for optimizing the sensitivity with the Vernier configurations is the length of the SCF. This length has to be chosen following practical considerations such as the temperature range to be measured and the bandwidth of the instrument used for spectral analysis. This analysis should thus be helpful to provide the necessary guidelines for constructing Vernier-based fiber optic sensors with optimal sensitivity for different sensing applications.

## Methods

### Normalized intensity of an SMF–SCF–SMF device

According to coupled-mode theory, the complex amplitude of the central core of an SCF of length *L* is given by^73,74^:

17 $$A={e}^{i\kappa L}\left[\mathrm{cos}\left(\sqrt{7}\kappa L\right)-\frac{i}{\sqrt{7}}\mathrm{sin}\left(\sqrt{7}\kappa L\right)\right],$$

Thus, the normalized intensity of the central core is:

18 $${\left|A\right|}^{2}=I={\mathrm{cos}}^{2}\left(\sqrt{7}\kappa L\right)+\frac{1}{\sqrt{7}}{\mathrm{sin}}^{2}\left(\sqrt{7}\kappa L\right).$$

Using the trigonometric identities ${\mathrm{cos}}^{2}\theta +{\mathrm{sin}}^{2}\theta =1$ and ${2\mathrm{ cos}}^{2}\theta -1=2 \mathrm{cos}2\theta$, the normalized intensity can be written as:

19 $$I=\frac{3}{7}\mathrm{cos}\left(2\sqrt{7}\kappa L\right)+\frac{4}{7}$$

### Envelopes of SCF Vernier spectra

For two SMF–SCF–SMF devices connected in series and with transmission readout, the normalized intensity is given by:

20 $${I}_{ser}^{Tx}=\frac{1}{49}\left\{3\mathrm{cos}\left(2\sqrt{7}{\kappa }_{s}{L}_{s}\right)+4\right\}\times \left\{3\mathrm{cos}\left(2\sqrt{7}{\kappa }_{r}{L}_{r}\right)+4\right\},$$

where the subscripts *r* and *s* indicate the reference and sensing devices, respectively. Expanding this product, we get:

$${I}_{ser}^{Tx}=\frac{1}{49}\left\{9\mathrm{cos}\left(2\sqrt{7}{\kappa }_{s}{L}_{s}\right)\mathrm{cos}\left(2\sqrt{7}{\kappa }_{r}{L}_{r}\right)+12\mathrm{cos}\left(2\sqrt{7}{\kappa }_{s}{L}_{s}\right)+12\mathrm{cos}\left(2\sqrt{7}{\kappa }_{r}{L}_{r}\right)+16\right\}.$$

Using $2cos\theta cos\varphi =cos\left(\theta -\varphi \right)+cos\left(\theta +\varphi \right)$, we can write the first term as the sum and the difference of the arguments and thus obtain:

$${I}_{ser}^{Tx}=\frac{1}{49}\left\{\frac{9}{2}\mathrm{cos}\left(2\sqrt{7}{\kappa }_{s}{L}_{s}-2\sqrt{7}{\kappa }_{r}{L}_{r}\right)+\frac{9}{2}\mathrm{cos}\left(2\sqrt{7}{\kappa }_{s}{L}_{s}+2\sqrt{7}{\kappa }_{r}{L}_{r}\right)+12\mathrm{cos}\left(2\sqrt{7}{\kappa }_{s}{L}_{s}\right)+12\mathrm{cos}\left(2\sqrt{7}{\kappa }_{r}{L}_{r}\right)+16\right\}.$$

$${I}_{ser}^{Tx}=\frac{1}{98}\left\{9\mathrm{cos}\left(2\sqrt{7}{\kappa }_{s}{L}_{s}-2\sqrt{7}{\kappa }_{r}{L}_{r}\right)+9\mathrm{cos}\left(2\sqrt{7}{\kappa }_{s}{L}_{s}+2\sqrt{7}{\kappa }_{r}{L}_{r}\right)+24\mathrm{cos}\left(2\sqrt{7}{\kappa }_{s}{L}_{s}\right)+24\mathrm{cos}\left(2\sqrt{7}{\kappa }_{r}{L}_{r}\right)+32\right\}.$$

We can also express the third and fourth terms as the sum and the difference of the arguments by using the trigonometric identity: $cos\theta +cos\varphi =2cos\left(\frac{\theta -\varphi }{2}\right)cos\left(\frac{\theta +\varphi }{2}\right)$. This substitution leads to:

$${I}_{ser}^{Tx}=\frac{1}{98}\left\{9\mathrm{cos}\left(2\sqrt{7}{\kappa }_{s}{L}_{s}-2\sqrt{7}{\kappa }_{r}{L}_{r}\right)+9\mathrm{cos}\left(2\sqrt{7}{\kappa }_{s}{L}_{s}+2\sqrt{7}{\kappa }_{r}{L}_{r}\right)+48\mathrm{cos}\left(\sqrt{7}{\kappa }_{s}{L}_{s}-\sqrt{7}{\kappa }_{r}{L}_{r}\right)\mathrm{cos}\left(\sqrt{7}{\kappa }_{s}{L}_{s}+\sqrt{7}{\kappa }_{r}{L}_{r}\right)+32\right\}$$

With this expression, we can start looking for the corresponding envelope (i.e., the low-frequency signal in which local maxima values occur). First, we can expect the first term to be part of the envelope as the argument is proportional to the length difference of the devices. Then, we can notice that the second term, $9\mathrm{cos}\left(2\sqrt{7}{\kappa }_{s}{L}_{s}+2\sqrt{7}{\kappa }_{r}{L}_{r}\right)$, corresponds to the first harmonic of the high-frequency component of the third term, $\mathrm{cos}\left(\sqrt{7}{\kappa }_{s}{L}_{s}+\sqrt{7}{\kappa }_{r}{L}_{r}\right)$. This relation means that for values of the argument in which the third term reaches a local maximum or minimum, the second term reaches its maximum. In other words, when $2\sqrt{7}{\kappa }_{s}{L}_{s}+2\sqrt{7}{\kappa }_{r}{L}_{r}=n\pi$, an extreme value is expected in ${I}_{ser}^{Tx}$. For this condition, the second term reduces to 9, and the high-frequency component in the third term is reduced to ± 48 as a maximum and minimum alternate. Using this, we can thus elaborate an expression for the envelope that reads:

21 $${E}_{ser}^{Tx}=\frac{1}{98}\left\{9\mathrm{cos}\left(2\sqrt{7}{\kappa }_{s}{L}_{s}-2\sqrt{7}{\kappa }_{r}{L}_{r}\right)+9\pm 48\mathrm{cos}\left(\sqrt{7}{\kappa }_{s}{L}_{s}-\sqrt{7}{\kappa }_{r}{L}_{r}\right)+32\right\},$$

Finally, grouping terms and defining $\alpha =\sqrt{7}{\kappa }_{s}{L}_{s}-\sqrt{7}{\kappa }_{r}{L}_{r}$, we obtain Eq. (2).

Similarly, for two SMF–SCF–SMF devices connected in parallel and with transmission readout, the normalized intensity is:

22 $${I}_{par}^{Tx}=\frac{1}{14}\left\{3\mathrm{cos}\left(2\sqrt{7}{\kappa }_{s}{L}_{s}\right)+4\right\}+\frac{1}{14}\left\{3\mathrm{cos}\left(2\sqrt{7}{\kappa }_{r}{L}_{r}\right)+4\right\},$$

$${I}_{par}^{Tx}=\frac{1}{14}\left\{3\mathrm{cos}\left(2\sqrt{7}{\kappa }_{s}{L}_{s}\right)+3\mathrm{cos}\left(2\sqrt{7}{\kappa }_{r}{L}_{r}\right)+8\right\},$$

Again, using $cos\theta +cos\varphi =2cos\left(\frac{\theta -\varphi }{2}\right)cos\left(\frac{\theta +\varphi }{2}\right)$, we obtain:

$${I}_{par}^{Tx}=\frac{1}{14}\left\{6\mathrm{cos}\left(\sqrt{7}{\kappa }_{s}{L}_{s}-\sqrt{7}{\kappa }_{r}{L}_{r}\right)6\mathrm{cos}\left(\sqrt{7}{\kappa }_{s}{L}_{s}+\sqrt{7}{\kappa }_{r}{L}_{r}\right)+8\right\},$$

Then, it is clear that the envelope of ${I}_{par}^{Tx}$ follows the low-frequency component $6\mathrm{cos}\left(\sqrt{7}{\kappa }_{s}{L}_{s}-\sqrt{7}{\kappa }_{r}{L}_{r}\right)$ which leads, substituting *α*, to Eq. (3).

For the case of series connection in reflection readout, the light passes twice through each device before recording the corresponding spectra. Thus, the normalized intensity is:

23 $${I}_{ser}^{Rx}=\frac{1}{{98}^{2}}\left\{3\mathrm{cos}\left(2\sqrt{7}{\kappa }_{s}{L}_{s}\right)+4\right\}\times \left\{3\mathrm{cos}\left(2\sqrt{7}{\kappa }_{r}{L}_{r}\right)+4\right\}\times \left\{3\mathrm{cos}\left(2\sqrt{7}{\kappa }_{s}{L}_{s}\right)+4\right\}\times \left\{3\mathrm{cos}\left(2\sqrt{7}{\kappa }_{r}{L}_{r}\right)+4\right\},$$

$${I}_{ser}^{Rx}={I}_{ser}^{Tx}\times {I}_{ser}^{Tx}$$

$${I}_{ser}^{Rx}={\left\{{I}_{ser}^{Tx}\right\}}^{2}$$

Then, the envelope can be obtained as:

24 $${E}_{ser}^{Rx}=\frac{1}{{98}^{2}}{\left\{9\mathrm{cos}\left(2\sqrt{7}{\kappa }_{s}{L}_{s}-2\sqrt{7}{\kappa }_{r}{L}_{r}\right)+9\pm 48\mathrm{cos}\left(\sqrt{7}{\kappa }_{s}{L}_{s}-\sqrt{7}{\kappa }_{r}{L}_{r}\right)+32\right\}}^{2},$$

Finally, for parallel connection in reflection readout, the normalized intensity is:

25 $${I}_{par}^{Rx}=\frac{1}{{2\times 7}^{2}}{\left\{3\mathrm{cos}\left(2\sqrt{7}{\kappa }_{s}{L}_{s}\right)+4\right\}}^{2}+\frac{1}{{2\times 7}^{2}}{\left\{3\mathrm{cos}\left(2\sqrt{7}{\kappa }_{r}{L}_{r}\right)+4\right\}}^{2},$$

$${I}_{par}^{Rx}=\frac{1}{98}\left\{9{\mathrm{cos}}^{2}\left(2\sqrt{7}{\kappa }_{s}{L}_{s}\right)+24\mathrm{cos}\left(2\sqrt{7}{\kappa }_{s}{L}_{s}\right)+16\right\}+\frac{1}{{7}^{2}}\left\{9{\mathrm{cos}}^{2}\left(2\sqrt{7}{\kappa }_{r}{L}_{r}\right)+24\mathrm{cos}\left(2\sqrt{7}{\kappa }_{r}{L}_{r}\right)+16\right\}$$

Now, using $${cos}^{2}\theta -\frac{1}{2}=\frac{1}{2}cos2\theta $$,

$${I}_{par}^{Rx}=\frac{1}{98}\left\{\frac{9}{2}\mathrm{cos}\left(4\sqrt{7}{\kappa }_{s}{L}_{s}\right)+24\mathrm{cos}\left(2\sqrt{7}{\kappa }_{s}{L}_{s}\right)+\frac{41}{2}\right\}+\frac{1}{{7}^{2}}\left\{\frac{9}{2}\mathrm{cos}\left(4\sqrt{7}{\kappa }_{r}{L}_{r}\right)+24\mathrm{cos}\left(2\sqrt{7}{\kappa }_{r}{L}_{r}\right)+\frac{41}{2}\right\}$$

$${I}_{par}^{Rx}=\frac{1}{98}\left\{\frac{9}{2}\mathrm{cos}\left(4\sqrt{7}{\kappa }_{s}{L}_{s}\right)+\frac{9}{2}\mathrm{cos}\left(4\sqrt{7}{\kappa }_{r}{L}_{r}\right)+24\mathrm{cos}\left(2\sqrt{7}{\kappa }_{s}{L}_{s}\right)+24\mathrm{cos}\left(2\sqrt{7}{\kappa }_{r}{L}_{r}\right)+41\right\}$$

Then, using again $cos\theta +cos\varphi =2cos\left(\frac{\theta -\varphi }{2}\right)cos\left(\frac{\theta +\varphi }{2}\right)$,

$${I}_{par}^{Rx}=\frac{1}{98}\left\{9\mathrm{cos}\left(2\sqrt{7}{\kappa }_{s}{L}_{s}-2\sqrt{7}{\kappa }_{r}{L}_{r}\right)\mathrm{cos}\left(2\sqrt{7}{\kappa }_{s}{L}_{s}+2\sqrt{7}{\kappa }_{r}{L}_{r}\right)+48\mathrm{cos}\left(\sqrt{7}{\kappa }_{s}{L}_{s}-\sqrt{7}{\kappa }_{r}{L}_{r}\right)\mathrm{cos}\left(\sqrt{7}{\kappa }_{s}{L}_{s}+\sqrt{7}{\kappa }_{r}{L}_{r}\right)+41\right\}$$

Using a similar analysis to that used for ${I}_{ser}^{Tx}$, the extreme values in ${I}_{par}^{Rx}$ are expected to occur when the local maxima in the first two terms coincide. As local maxima are governed by the high-frequency cosine factors and owning the harmonic nature of the two first terms, the local maxima coincide when $2\sqrt{7}{\kappa }_{s}{L}_{s}+2\sqrt{7}{\kappa }_{r}{L}_{r}=n\pi$. For these cases, the high-frequency function of the first term,$\mathrm{cos}\left(2\sqrt{7}{\kappa }_{s}{L}_{s}+2\sqrt{7}{\kappa }_{r}{L}_{r}\right),$ equals 1, while the corresponding high-frequency function of second terms,$\mathrm{cos}\left(\sqrt{7}{\kappa }_{s}{L}_{s}+\sqrt{7}{\kappa }_{r}{L}_{r}\right),$ equals ± 1. Thus, an expression for the envelope of ${I}_{par}^{Rx}$ may be written as:

26 $${E}_{par}^{Rx}=\frac{1}{98}\left\{9\mathrm{cos}\left(2\sqrt{7}{\kappa }_{s}{L}_{s}-2\sqrt{7}{\kappa }_{r}{L}_{r}\right)\pm 48\mathrm{cos}\left(\sqrt{7}{\kappa }_{s}{L}_{s}-\sqrt{7}{\kappa }_{r}{L}_{r}\right)+41\right\}.$$

Interestingly, although the expressions for ${I}_{ser}^{Tx}$ and ${I}_{par}^{Rx}$ are essentially different, the envelopes found under this analysis are identical for these two cases.

### Construction of SMF–SCF–SMF devices

The devices used in our experiments were fabricated upon fusion splicing the SMF and the SCF. After performing the first splice to join both fibers, the SCF was mounted over a fiber cleaver and displaced to the desired length using a translational stage with a micrometer. The second end of the SCF segment was finally spliced to an SMF. It should be noted that the fabrication process of these fiber devices does not require chemical etching, fiber tapering, or laser processing, and only commercially available equipment is needed.
